# Analysis of human alveolar osteoblast behavior on a nano-hydroxyapatite substrate: an *in vitro* study

**DOI:** 10.1186/1472-6831-14-22

**Published:** 2014-03-20

**Authors:** Andrea Pilloni, Giorgio Pompa, Matteo Saccucci, Gabriele Di Carlo, Lia Rimondini, Marina Brama, Blerina Zeza, Francesca Wannenes, Silvia Migliaccio

**Affiliations:** 1 Department of Oral and Maxillofacial Science, Periodontics Unit, Sapienza University of Rome, Rome, Italy; 2 Department of Oral and Maxillofacial Science, Prosthodontics Unit, Sapienza University of Rome, Rome, Italy; 3 Department of Oral and Maxillofacial Science, Pediatric Dentistry Unit, Sapienza University of Rome, Rome, Italy; 4 Department of Health Sciences, Laboratory of Biomedical and Dental Materials, University of Oriental Piedmont “Amedeo Avogadro”, Novara, Italy; 5 Medical Phatophysiology, Endocrinology and Nutrition Unit, Department of Experimental Medicine, Sapienza University of Rome, Rome, Italy

**Keywords:** Nanohydroxyapatite, Human osteoblasts, Alveolar bone regeneration

## Abstract

**Background:**

Nano-hydroxyapatite (nHA) is a potential ideal biomaterial for bone regeneration. However, studies have yet to characterize the behavior of human osteoblasts derived from alveolar bone on nHA. Thus, the aim of the present study was to evaluate the influence of nHA on the adhesion, proliferation and differentiation of these alveolar bone-derived cells.

**Methods:**

Primary human alveolar osteoblasts were collected from the alveolar ridge of a male periodontal patient during osseous resective surgery and grown on culture plates coated with either polylysine or polylysine with nano-hydroxyapatite (POL/nHA) composite. The cells were grown and observed for 14 days, and then assessed for potential modifications to osteoblasts homeostasis as evaluated by quantitative reverse transcriptase-polymerase chain reaction (real time RT-PCR), scanning electron microscopy and atomic force microscopy.

**Results:**

Real time PCR revealed a significant increase in the expression of the selected markers of osteoblast differentiation (bone morphogenetic protein (BMP)-2,-5,-7, ALP, COLL-1A2, OC, ON) in cells grown on the POL/nHA substrate. In addition, as compared with the POL surface, cells grown on the POL/nHA substrate demonstrated better osteoconductive properties, as demonstrated by the increase in adhesion and spreading, likely as a result of the increased surface roughness of the composite.

**Conclusions:**

The increased expression of BMPs and osteoinductive biomarkers suggest that nano-hydroxyapatite may stimulate the proliferation and differentiation of local alveolar osteoblasts and thus encourage bone regeneration at sites of alveolar bone regeneration.

## Background

The main concern of periodontology is the rehabilitation of periodontally compromised teeth, possibly aiming at the complete periodontal tissue regeneration [1]. Ideally, this process would include the formation of new bone, new periodontal ligament and new cementum, or rather a new attachment over the root that has previously been deprived of all the attachment apparatus [2]. The therapeutic approaches attempting to achieve this objective include the use of different grafting materials (autogenous, allogeneic, xenogeneic and alloplastic grafts), physical barriers for Guided Tissue Regeneration (GTR), enamel matrix-derived proteins, and various growth factors [3,4].

It is assumed that the use of bone grafts or alloplastic materials would result both in the regrowth of alveolar bone and the formation of a new cementum layer with inserted collagen fibers onto the previously periodontitis-involved root surface [5]. The mechanism of action may be either via the stimulation of osteogenesis (new bone formation from the bone-forming cells contained in the graft), osteoconduction (when the graft serves as a scaffold for bone formation from the adjacent host bone), or osteoinduction (the matrix of the bone graft contains bone-inducing substances that result in bone formation in the surrounding tissues even if not bone tissue) [6]. Alloplastic materials are presumed to promote bone healing through osteoconduction and subsequently behave as a scaffold for this process [5].

An ideal scaffold is a biocompatible material that provides appropriate mechanical support [7]. It should possess a structure similar to the native extracellular matrix, exhibit favorable surface properties, and lead to an increase in adhesion, proliferation and differentiation of osteoblastic cells [7]. Hydroxyapatite (HA) [Ca_5_ (PO_4_)_3_ OH] is an alloplastic material, chemically similar to the inorganic component of bone matrix that translates these properties in a valuable and optimal biocompatibility [7]. When HA is grafted beneath a healthy periosteum and well-vascularized bone, it first becomes integrated by a clot [8] and then releases phosphate ions into the surrounding environment, which stimulates neo-osteogenesis.

A fundamental factor governing optimal integration of HA with bone is the dimensions of the crystals. HA particles with dimensions closer to the size of natural crystals found in vertebrate hard tissues (i.e., ranging from 1 to 10 nm), are now available [9], and they have been reported to mimic the extracellular matrix of bone in size and structure [10]. *In vitro* studies conducted with the purpose of understanding the mechanism of action of this nano-sized material have described a stimulatory effect on mesenchymal stem cells [11], an increase in protein absorption and osteoblast adhesion as compared with traditional micro-sized HA [12,13], stimulation of human osteoblast-like cell proliferation resulting in bone formation [14], and the stimulation of PDL cell attachment [15]. Despite this, there is little information to date regarding the responses of human alveolar osteoblasts in contact with this grafting material once the bone defect is filled. Thus, the aim of our study was to evaluate *in vitro* the effects of nHA on the adhesion and proliferation of human alveolar osteoblasts and to determine the impact that this alloplastic material has on the expression of biomarkers of bone formation during HA-mediated periodontal bone regeneration.

## Methods

### Isolation of human primary osteoblasts

Fresh human bone removed from the alveolar crest were obtained with approval from the Ethical Committee of Sapienza University of Rome and informed patient consent.

Primary human alveolar osteoblasts (hOBs) were harvested from a 53-year-old male periodontal patient during resective periodontal surgery according to a previously validated protocol [16,17].

Harvested bone specimens were stored in Hank’s Balanced Salt Solution (HBSS, Sigma-Aldrich, St. Louis, MO) containing 100 U/ml penicillin and 100 mg/ml streptomycin until processing. For processing, bone samples were rinsed in sterile phosphate-buffered saline (PBS), pH 7.4, fragmented with a scalpel, and digested in solution of 1 mg/ml type IV collagenase and 0.25% trypsin (in PBS) at 37°C for 30 min with agitation, followed two additional digestion stages for 40 min and 90 min, as previously described [18,19]. Cells obtained from the second and third digestions were collected, centrifuged and resuspended in Dulbecco’s Modified Eagles Medium (DMEM, Gibco-BRL, Rockville, MD) supplemented with 50 U/ml penicillin, 50 μg/ml streptomycin, 4 mmol/l L-glutamine and 10% of fetal bovine serum (FBS, Hyclone, Thermo Scientific, Wilmington, DE). Cells adhesion, proliferation and morphology were visually inspected by microscopy (Nikon Eclipse TS100, Nikon, Tokyo, Japan) over the 2-week time frame. After the cells reached 70-80% confluence, they were detached with trypsin-EDTA, harvested and used for experiments between passages 2 and 4.

### Poly-L-lysine and Poly-L-lysine-nHA coating preparation

One milliliter per 25 cm^2^ of 0.01% poly-L-lysine solution (POL, Bioreagent, mol wt 150,000-300,000, sterile-filtered, Sigma-Aldrich) was used to coat the surfaces of Petri plates. Samples were gently shaken at 4°C at 1200 rpm for 10 min to homogenize stratification. The surfaces were then rinsed with sterile water and allowed to dry at room temperature. Subsequently, 10 mg of nHA powder (Ghimas Spa, Casalecchio di Reno, BO, Italy) was resuspended in 1 ml of sterile water and used to coat the POL-treated plates. Finally, coated surfaces were rinsed with sterile water and allowed to dry.

### Osteoblasts-POL and osteoblasts-POL-nHA samples preparation

Osteoblast behavior was examined on coated plates using two tests: in Test 1, plates coated only with POL were seeded with hOBs onto at a density of 2 × 10^4^ cells/cm^2^; in Test 2, hOBs were seeded at the same density onto plates coated with POL and nHA powder. Samples of Test 1 and Test 2 were cultured under the same experimental conditions (95% humidity, 5% CO_2,_ 37°C) in DMEM containing 10% FBS. Experiments were performed in triplicate and repeated three times each.

### Scanning electron microscopy

Surface properties of the coated plates and osteoblast morphology were visually investigated by scanning electron microscopy (SEM). After 24 h of culture on POL or POL-nHA coated surfaces, samples were rinsed twice with PBS, fixed in 2.5% glutaraldehyde for 1 h, rinsed with distilled water, dehydrated with graded concentrations of alcohol (50%, 70%, 90%, 100% ethanol) and finally treated with CO_2_ at the top-critical point. Specimens were fixed onto aluminum stubs using a conductive carbon tape and then coated with a 10-nm gold layer (Coating Unit E5000, Polaron Ltd. Watford, UK). Specimens were observed with SEM (Leo 1450VP, Carl Zeiss, Jena, Germany) at various magnifications, using secondary electrons at 15 kV.

### Atomic force microscopy

Atomic force microscopy (AFM) was carried out using a Nanonics Imaging AFM system (Jerusalem, Israel) in a no contact mode and a reference voltage of 0.8–1.0 V between the tip and the sample surface during all experiments.

### Gene expression

After 14 days of culture on POL and POL-nHA surfaces, RNA was extracted according to the protocol previously described [18]. RNA was reversed transcribed using TaqMan reverse transcription kit (Applied Biosystems, Life Technologies, Foster City, CA), using random hexamers. Real time PCR was then performed using SYBR green gene expression assay (Applied Biosystems) on an ABI Prism 7300 Real Time PCR system (Applied Biosystems) to assess for changes in the expression of osteogenic markers: alkaline phosphatase (ALP), A2 pro-collagen type 1 chain (COLL-1A2), bone morphogenetic protein (BMP)-2, BMP-5, BMP-7, osteocalcin and osteonectin. Glyceraldehyde-3 phosphate-dehydrogenase (GAPDH) was used as an endogenous control. Sequences were designed using Primer Express software (Applied Biosystems) and synthesized by Primm (Milan, Italy). Primers are listed in Table 1. Gene expression was analyzed according to the ^ΔΔ^CT method, with the expression levels of Test 2 (POL/nHA) samples normalized towards Test 1 (POL) values.

**Table 1:** Human primers used in RT-PCR for Glyceraldehyde-3 phosphate-dehydrogenase (GAPDH), alkaline phosphatase (ALP), A2 pro-collagen type 1 chain (COLL-1A2), bone morphogenetic protein-2,-5,-7 (BMP-2,-5,-7)

**Target gene**	**Primer sequences**
GADPH	5′-GGAGTCAACGGATTTGGTCGTA-3′
	5′-GGCAACAATATCCACTTTACCAGAGT-3′
ALP	5′-TGCGGAAGAACCCCAAAG-3′
	5′-ATGGTGCCCGTGGTCAAT-3′
Coll-1A2	5′-CCCAGCCAAGAACTGGTATAGG-3′
	5′-GGCTGCCAGCATTGATAGTTTC-3′
Osteocalcin	5′-AGCAAAGGTGCAGCCTTTGT-3′
	5′-GCGCCTGGGTCTCTTCACT-3′
Osteonectin	5′-CCACACGTTTCTTTGAGACC-3′
	5′-GATGTCCTGCTCCTTGATGC-3′
BMP-2	FW5′-CCAGCTGTAAGAGACACCCTTTG-3′
	RV5′-ACCCACAATCCAGTCATTCCA-3′
BMP-5	FW5′-CGTCCTCTGCACCTCTCTTTATG-3′
	RV5′-CTCCGACTCTTCAGGATTTTCTTC-3′
BMP-7	FW5′-TCTTCCACCCACGTACCA-3′
	RV5′-GCTTCCCCTTCTGGGATCTT-3′

### Statistical analysis of data

The Statistical Package for Social Sciences (SPPS v15.0, SPSS Inc. Chicago, IL) was used for statistical analysis. Analyses were performed using Student’s *t*-test. Significance was set at p < 0.05.

## Results

### Human primary osteoblasts extraction and culture

**Figure 1:**
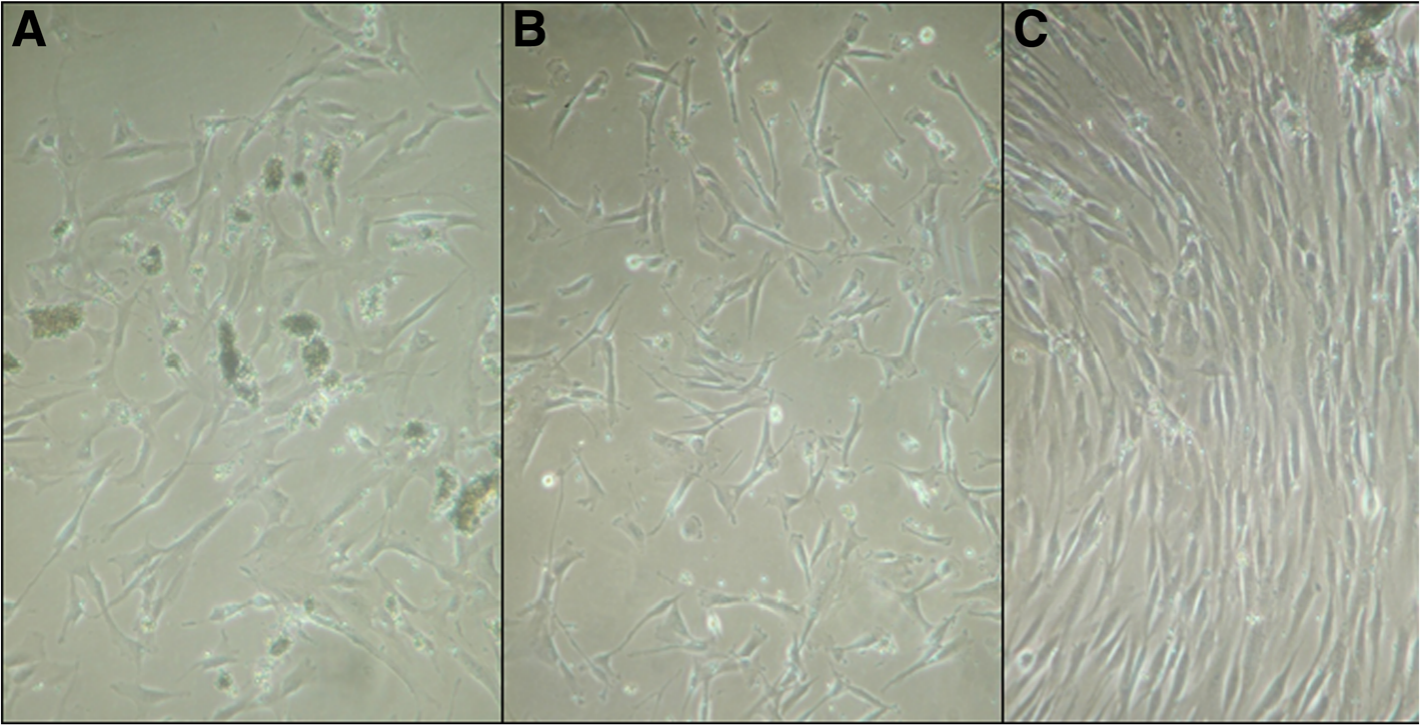
**Human primary osteoblasts. (A)** After 4 **(B)** After 5 days **(C)** After 14 days (Original magnification, 20×).

Osteoblasts successfully attached to the substrate surfaces and were observed to undergo proliferation from day 4 to day 14, as determine by visual inspection by microscopy (Figure 1A–C).

### POL and POL-nHA surface roughness properties

**Figure 2:**
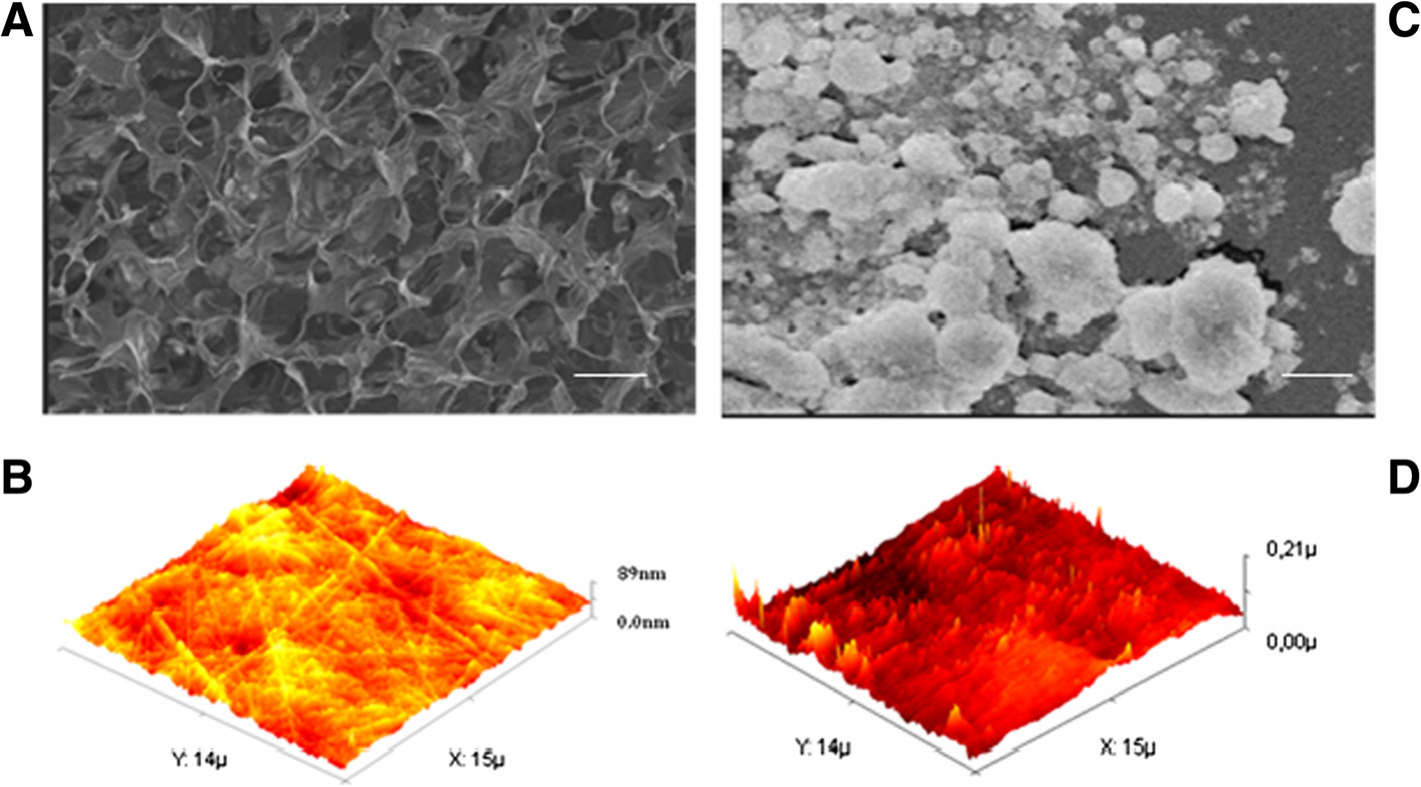
**Scanning microscopy. (A)** Electron microscopy image of the polylysine (POL) substrates. **(B)** Atomic force microscopy of POL/nanohydroxyapatite. **(C)** Scanning electron microscopy. **(D)** Atomic force microscopy of POL/nanohydroxyapatite (POL/nHA).

The roughness of both substrate surfaces was evaluated by SEM and AFM. The nanometric surface roughness evaluated by AFM was 49.7 rms/nm for POL-nHA and 6.3 rms/nm for POL. Thus, plates covered in POL-nHA showed a 7.89-fold higher nanometric superficial roughness compared with POL plates (Figure 2A-D). Additionally, SEM observations revealed the morphology of the nHA particles to have a rod-like shape of 70–90 nm in diameter.

### Effect of nano-hydroxyapatite on osteoblast adhesion and proliferation

**Figure 3:**
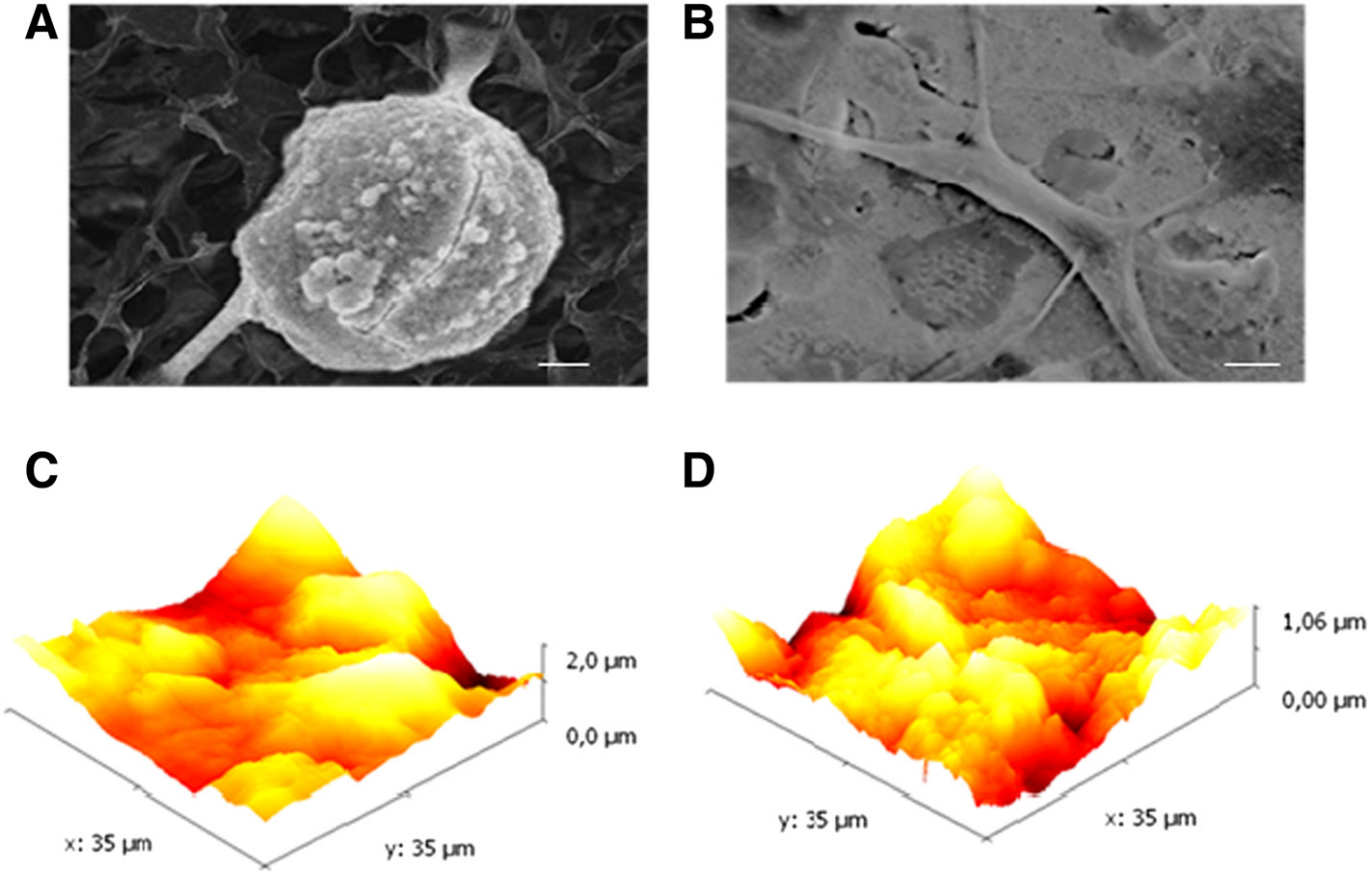
**Scanning electron microscopy (SEM) and atomic force microscopy (AFM). (A)** Cells grown on POL appeared spherical with retracted fibers. **(B)** Cells grown on POL/nHA appeared elongated and with many filopodia. **(C)** Cells grown on POL appeared spherical with retracted fibers. **(D)** Cells grown on POL/nHA appeared elongated and with many filopodia.

The potential morphological modifications to hOBs were evaluated after the cells were seeded onto plates coated with POL and POL-nHA. After 24 h, cells growing on the POL-coated plates maintained a spherical form (Figure 3A), whereas cells on the POL-nHA substrate appeared to be attached firmly, as demonstrated by the presence of numerous filopodia extensions (Figure 3B), suggesting that the nHA particles improved osteoblast adhesion and spreading. Moreover, images obtained by AFM showed that hOBs cultured on POL/nHA adhered stronger than those on POL alone, as shown by the increase in nanometric superficial roughness (Figure 3C-D).

### Effects of nano-hydroxyapatite on gene expression of human osteoblasts

**Figure 4:**
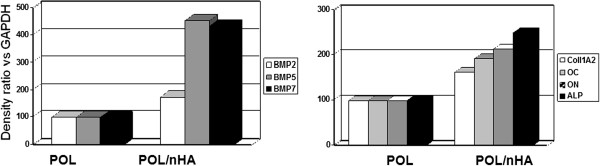
Gene expression of hOBs placed in culture in POL and POL/nHA.

RNA was extracted from cells cultured for 14 days on both substrates (POL versus POL/nHA) and subjected to real time RT-PCR to assess for changes in the expression of specific osteoblastic differentiation genes (Table 1): BMP-2, -5 and -7, ALP, COLL-1A2, OC and ON, well-known differentiation markers. As shown in Figure 4, BMP-2, BMP-5 and BMP-7 were increased by approximately 1.7, 4.5 and 4.3 times, respectively, in hOBs cultured on POL/nHA substrates as compared with those cultured on POL alone (p < 0.05). Interestingly, COLL-1A2, OC and ON expression was 1.6, 1.9 and 2.1 times higher, respectively, in hOBs grown on POL/nHA as compared with those grown on POL (p < 0.01). ALP was also increased 2.5 times in hOBs grown on POL/nHA (p < 0.01), suggesting an increase in differentiation of these cells under the current culture conditions.

Overall, our results show that, within a 14-day time frame, POL/nHA substrate can enhance the expression of specific osteoblastic genes, which strongly indicates a positive effect of nHA on osteoblast differentiation (Figure 4).

## Discussion

Hydroxyapatite, among the various bone grafting substitutes, has been widely investigated in periodontal regeneration over the past three decades [4,20]. HA alloplastic grafts, despite their similar chemical composition to bone, are known mainly for their osteoconductive properties [5]. Our study has confirmed the osteoconductive properties of a nano-sized version of this material, showing that nHA can stimulate osteoblasts to produce selected biomarkers representative of bone formation and mesenchymal cell recruitment.

The rationale for the choice of human alveolar osteoblasts is based on the fact that these cells are located on the bone surface of the defect, meaning that the grafted material will be in direct contact with these osteoblasts during healing. The cell collection method was performed using a bone scalpel as part of a well-documented technique used during resective periodontal surgery [21,22]. Cell culture and formation of the substrates were performed followed models previously used (dispersion of particles of nHA in poli-ethylene-glicol-dimethacrylate or nHA associated with a polycarbonate, polyamide, polysaccharide) [23-27]. Human alveolar osteoblasts are the primary cells responsible for alveolar bone formation, although they do not appear to be able to migrate and proliferate at distant sites where bone regeneration is required. Albrektsson & Johansson suggested that pre-existing osteoblastic cells only contribute a minor portion of the new bone needed in a fracture healing situation and that the recruitment of immature cells and their differentiation into osteoblasts are probably the basic and pivotal mechanism regulating bone healing [6]. Indeed, osteoblasts have been shown to contribute to the production of growth factors that encourage cellular migration as well as the osteogenic and chondrogenic differentiation of mesenchymal progenitor cells [28]. Our study demonstrates that nHA stimulates the local alveolar osteoblasts to produce relevant bone-specific BMPs, which are known to initiate and regulate bone formation starting from the progenitor cells.

Several types of BMPs, which belong to the transforming growth factor (TGF)-β family, have been investigated and characterized for their modulatory role in bone tissue homeostasis. BMP-2 and BMP-7 are of particular interest, since they are naturally released in response to trauma or during bone remodeling and are, to date, the only known inductive agents [6]. Stimulation of BMP-2, -5 and -7 is an important aspect of osteogenesis, as these proteins are the three of the most potent growth factors enhancing bone formation. The results presented herein show that nHA promotes BMP expression by alveolar osteoblasts *in vitro*. This observation suggests that nHA might act as promoter of bone regeneration at the bone defect site in two ways: by inducing osteogenic differentiation as observed by Lock et al. [11] and/or by inducing the secretion of specific factors, such as BMPs or other growth factors not tested in this experiment. Moreover, the nHA influenced a significant increase in the expression of several important markers of osteogenesis ALP, OC, ON and COLL-1A2.

To instigate bone formation, cells need to adhere and proliferate. Surface roughness is an important consideration when selecting a bone-inducing substrate. According to physical and mechanical principles, a substrate with superficial roughness shows a higher capacity for adhesion when compared with a smooth surface [29-32]. In this study, osteoblast adhesion was evaluated based on changes to the cell form and on the presence of filopodia. SEM and AFM images showed that nHA had a rougher surface than the control substrate and favored the adhesion and proliferation of human osteoblasts. These results are in agreement with those reported by Thian et al., showing that nHA enhanced adhesion and spreading of human osteoblast-like cells [33] and Liu et al., demonstrating the induction of adhesion and proliferation of human osteoblast-like MG-63 cells on nHA [14].

## Conclusions

Within the limits of this study, we observed that nHA can significantly increase on site human alveolar osteoblast adhesion and differentiation, and therefore speculate that nHA could encourage bone formation by local osteoblasts at sites of alveolar bone reconstruction. In addition, the increased expression of BMPs on the nHA surface could indicate an increase in the recruitment of progenitor cells during healing with nHA scaffolds. These *in vitro* findings could explain, at least in part, the new bone formation witnessed in bone defects filled with nHA grafting material [34,35] and suggest the further role of nHA in periodontal reattachment processes, including the formation of new cementum and new periodontal ligament. Further histological and clinical studies are needed to give significance to the *in vitro* results as well as a biological explanation of the clinical outcomes observed by others.

## Competing interests

The authors declare that they have no competing interests.

## Authors’ contributions

AP and SM were the principal investigators of this study. They made substantial contributions to conception and design, as well as the acquisition, analysis and interpretation of data; GP, MS, GDC and BZ were involved in drafting the manuscript or revising it critically for important intellectual content, and gave final approval of the version to be published. LR, MB and FW made substantial contributions in acquisition of data and participated in drafting the manuscript and helped in the revision of the manuscript. All authors read and approved the final manuscript.

## Pre-publication history

The pre-publication history for this paper can be accessed here:

http://www.biomedcentral.com/1472-6831/14/22/prepub
